# A Drug-Centric View of Drug Development: How Drugs Spread from Disease to Disease

**DOI:** 10.1371/journal.pcbi.1004852

**Published:** 2016-04-28

**Authors:** Raul Rodriguez-Esteban

**Affiliations:** Roche Pharmaceutical Research and Early Development, pRED Informatics, Roche Innovation Center, Basel, Switzerland; Philadelphia, UNITED STATES

## Abstract

Drugs are often seen as ancillary to the purpose of fighting diseases. Here an alternative view is proposed in which they occupy a spearheading role. In this view, drugs are technologies with an inherent therapeutic potential. Once created, they can spread from disease to disease independently of the drug creator’s original intentions. Through the analysis of extensive literature and clinical trial records, it can be observed that successful drugs follow a life cycle in which they are studied at an increasing rate, and for the treatment of an increasing number of diseases, leading to clinical advancement. Such initial growth, following a power law on average, has a degree of momentum, but eventually decelerates, leading to stagnation and decay. A network model can describe the propagation of drugs from disease to disease in which diseases communicate with each other by receiving and sending drugs. Within this model, some diseases appear more prone to influence other diseases than be influenced, and vice versa. Diseases can also be organized into a drug-centric disease taxonomy based on the drugs that each adopts. This taxonomy reflects not only biological similarities across diseases, but also the level of differentiation of existing therapies. In sum, this study shows that drugs can become contagious technologies playing a driving role in the fight against disease. By better understanding such dynamics, pharmaceutical developers may be able to manage drug projects more effectively.

## Author Summary

The number of drug-like chemical and biological substances that can be constructed with current technologies is vast, yet the subset that can become an effective drug is far more restricted due to the many design constraints and the expense of development. Once developed, however, a drug can have multiple biological effects that can be beneficial in a variety of diseases. Here, it is shown that this combination of scarcity and polyvalence leads some drugs to propagate from disease to disease in a contagious manner. This analysis offers an alternative view of the drug development process in which drugs are central and can define dynamic relationships between diseases.

## Introduction

While drug development is typically thought of as the disease-centric process of finding a drug that can treat a disease, much effort goes in the reverse, drug-centric direction of finding a disease that can be treated by a drug. The diseases for which a drug is intended can change over the course of its development and post-marketing (see, for example, the case of tamoxifen [1]). During pharmaceutical development, new diseases can be selected or dropped at every stage of the pipeline on the basis of pre-clinical and clinical results. When a drug starts to show signs of success with a particular disease, additional diseases are sought to broaden the drug’s therapeutic and commercial appeal. Once a drug has been approved by regulatory agencies, its use may not be restricted to the diseases for which it was approved, as medical practitioners may prescribe it off-label [2]. Indeed, a drug’s efficacy against certain diseases may only become fully apparent once it is consumed by a large number of patients or made widely available for scientific experimentation. New findings about a drug’s efficacy can prompt the original drug developer to seek supplemental indication approvals or pursue life-cycle management strategies such as combining the drug with other new or existing drugs [3].

This is not to say that drugs are created *ex nihilo*. They are generally designed with an intent rooted in biological rationale, such as to inhibit a disease-causing gene. However, the interconnected nature of human biology and of pathological mechanisms, the steady advance in our understanding of diseases and the potential lack of target selectivity means that drugs designed for a specific purpose can end up having different or additional applications. Once a drug is created it can fail with diseases for which it was designed and succeed with unanticipated diseases. Thus, drugs hold an intrinsic value based not only on their proven therapeutic effect but also on their therapeutic *potential*, both suspected and unsuspected.

Because the process of pharmaceutical drug discovery is long and uncertain, a central part of a drug’s suspected therapeutic potential is the drug’s prospects to treat multiple diseases. Challenges to a drug’s development may come from faster-advancing competing drugs that can become standard of care and discourage further work on other drugs. They may also come from business vagaries such as department closures in pharmaceutical companies that lead to re-alignment of internal drug portfolios. Thus, having multiple potential applications increases the likelihood that a drug will be able to navigate the development process.

The unsuspected therapeutic potential of a drug is illustrated most clearly by the field of drug repurposing. Drug repurposing has drawn attention in part due to the commercial interest of pharmaceutical companies possessing an abundance of safe drugs that have failed to show sufficient efficacy in any disease. The “poster child” of drug repurposing success is that of a safe but abandoned drug that is discovered to be efficacious with a previously unsuspected disease [4], [5]. While drug repurposing focuses on late-stage and post-marketed drugs, the search for unsuspected diseases for existing drugs can be undertaken at any point in a drug’s history.

To further understand the process of uncovering the therapeutic potential of drugs, in this study I looked quantitatively at how drugs are matched with diseases. Quantitative analyses of drug-disease relationships have long been of interest, for example for drug safety [6] and repurposing [7], [8]. However, no studies have been performed on how drugs become paired with diseases. The closest prior work concerns the static network of diseases and their approved drugs [9–11], also called drug-therapy network or drug-disease network. The present study differs from that work in that the dynamic process of pairing drugs and diseases was analyzed over the course of time and in that every studied drug-disease pair, regardless of regulatory approval status, was considered.

## Results

To achieve broad coverage, two extensive datasets of clinical trials and scientific literature were employed. For the scientific literature, Medline records (*Literature* dataset) annotated with established drug names by a text mining algorithm (see Methods) were used. For clinical trials, records from ClinicalTrials.gov (*Clinical* dataset) were mapped to chemical and disease names (see Methods). While these datasets were rich in content, they had some limitations which are discussed below.

For simplicity of exposition certain language conventions are used throughout this text. In particular, drugs and diseases are personified. A drug’s “birth” is the first time a drug appears in a dataset and its “age” the time elapsed after its birth. (Note that in drug safety a drug’s birth date is, instead, the date of the first marketing authorization.) A “cohort” of drugs encompasses all drugs born in the same year. Drugs “accumulate” studies as they age, meaning that the total count of studies published about them increases over time. In the same fashion, drugs accumulate diseases over time as they are tested in additional diseases. A disease “adopts” a drug the first time the disease is paired with the drug.

**Fig 1:**
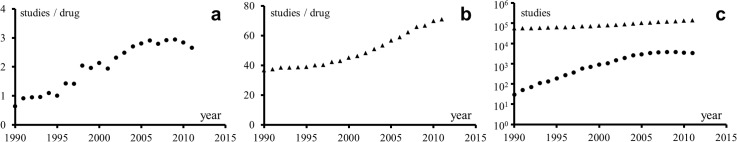
Average number of studies per drug per year. (a) *Clinical*. (b) *Literature*. (c) Number of studies in each dataset per year: *Literature* (triangles), *Clinical* (circles).

To analyze the relationship between the number of drugs and the number of studies that are performed about them, I counted the number of studies and the number of unique drugs mentioned each year in each of the datasets and looked at the ratio between these two quantities. As can be seen in Fig 1, there is a trend towards this ratio increasing in both datasets. Thus, each drug has been receiving greater attention over time, perhaps due to drugs becoming a relatively scarcer commodity.

However, there was high variability in the number of studies accumulated by individual drugs. In particular, there was high inequality within each cohort of drugs, although not reaching Pareto distribution levels due to the fact that the “richest” drugs did not accumulate enough studies. Nonetheless, the “richest” quintile of drugs from the 1990–1999 cohorts garnered, on average, 69% of the studies accumulated by each of those cohorts in the *Literature* dataset and 65% in the *Clinical* dataset. Not surprisingly, the number of studies accumulated by an individual drug was correlated with clinical advancement. For example, in the *Clinical* dataset, the drugs from the 1990–1999 cohorts that reached higher phases accumulated more studies on average. Drugs from the 1990–1999 cohorts that only reached phase I clinical trials by 2010 were on average at the 9th percentile of their cohort in number of studies accumulated, while those reaching phase II, III and IV were at the 20th, 39th and 63rd percentile, respectively.

**Fig 2:**
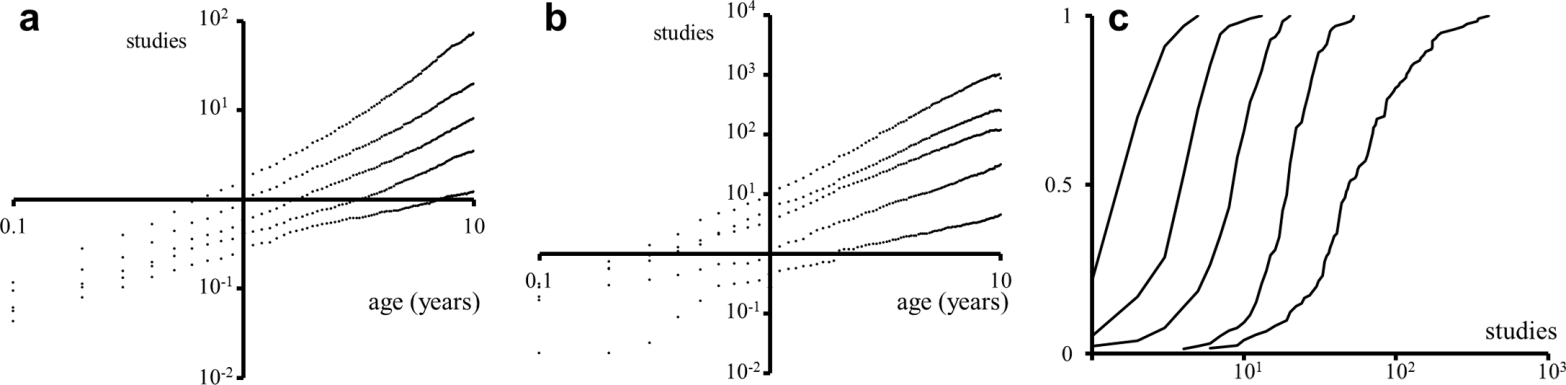
Average cumulative number of studies for drugs born during the period 1990–2010. (a) *Clinical*, (b) *Literature*. Each of the five trends in each chart corresponds to a quintile of the dataset, with the most studied drugs at the top and the least studied at the bottom (excluding the first study for each drug). The abscissa represents the age of the drug in years. As would be expected, the value of *β* increases for each quintile in each dataset. To construct the quintiles, drugs born in the same year were treated as a cohort. Each drug within a cohort was sorted into a quintile according to the number of studies it had accumulated by 2010. Thus, for example, drugs born in 1998 were compared only to drugs born in 1998 and not to older or newer drugs. Drugs studied only once were excluded. Regression curves for every trend were power laws with, respectively, the following *β* and *R*^*2*^ values, from upper quintile to lower quintile: (a) *Clinical*, *β* = {1.52, 1.25, 1.11, 1.09, 0.69} and *R*^*2*^ = {0.98, 0.99, 0.99, 0.93, 0.96}, (b) *Literature*, *β* = {1.95, 1.59, 1.45, 1.29, 1.13} and *R*^*2*^ = {0.99, 0.99, 0.99, 0.99, 0.96}. (c) Cumulative distribution of studies for every quintile at age 10 (left to right: lowest to highest quintile) for the *Clinical* dataset.

To analyze the temporal dynamics of this accumulation, I looked at the average number of studies that individual drugs accumulated over time and noted that the differences in the number of studies accumulated by each drug grew quickly over time. As can be seen in Fig 2, the average number of studies accumulated by drugs born between 1990 and 2010 followed a power law trend of the form *F*(*t*) = *αt*^*β*^ as they aged. This power law appeared for every cohort as well as for subsets of drugs, such as the ones shown in Fig 2 (in many cases a quadratic trend was also a good approximation). The sets of drugs that accumulated more studies were associated with both higher *α* and *β* values. A power law appeared regardless of the time window studied, albeit the time window chosen affected the resulting values of *α* and *β*. It is worth noting that the power law is based on the average accumulation and does not reflect the underlying variability, as can be seen in Fig 2C. Part of this variability comes from the fact that there has been an increase in the number of studies performed per drug over the years. Drugs also accumulated diseases following patterns of the form *αt*^*β*^. Heretofore the analysis focuses on the accumulation of studies by drugs but analogous results apply to the accumulation of diseases by drugs.

The derivative of the cumulative number of studies *F(t)* is the rate of studies, *F*′(*t*) = *αβt*^*β*−1^. As can be seen in Fig 2, decelerating *(β < 1*) trends are associated with drugs that have not accumulated many studies. At the most unsuccessful level, there are drugs that have only been studied once (and, of course, there is the universe of drugs that have never been studied). A value of *β > 1* corresponds to acceleration in the rate of studies, often corresponding to drugs that have met some success in the clinic. This can be observed in the *Literature* dataset, which largely consists of successful, established drugs which exhibit values of *β > 1*.

An interesting question is whether an accelerating trend (*β > 1*) can be sustained over the long term beyond the intervals of time considered here (≤ 20 yrs). Some such lasting trends are, in fact, found in the *Literature* dataset. These might be due to the fact that some drugs from the Literature dataset are especially successful and also due to the comparatively lower average cost of the studies described in *Literature* vs. the average cost of *Clinical* studies. Unfortunately, the *Clinical* dataset lacks enough historical depth for time windows beyond ~20 years but shorter-term trends can be nonetheless analyzed. I looked at *Clinical* drugs from the 1990–1999 cohorts and chose those at the top quintile (top 20%) in number of studies accumulated by age 5 (in comparison with drugs from their same cohorts). I then divided this group further into two halves according to their performance between ages 6 and 10. The drugs in the lowest-performing half went from having a *β* of 1.39 between birth and age 5 to having a *β* of 1.01 between ages 6 and 10. The highest performing half, on the other hand, went from a *β* of 1.60 to a *β* of 1.19. Thus, some of the “5-year-old top performers” followed five years of accelerated growth with a period of stagnating rate of growth or deceleration (*β < 1*). Therefore, even initially successful drugs can reach a point by which interest in further clinical development starts to stagnate and dwindle.

That said, certain degree of growth stability can be observed. Sixty-two per cent of 5-year-old upper-quintile performers were also upper-quintile performers between ages 6 and 10, while only 21% descended to the second quintile. Such “momentum” or, in economic terms, “lack of social mobility” could also be observed for drugs from the 1990–1999 cohorts in the *Literature* dataset, with 60% staying at the top quintile and 32% descending one level. Thus, it can be said that the development of successful drugs presents certain “inertia” or “momentum.” As in the driving of a heavy vehicle, it takes time both to accelerate and decelerate. In fact only a few upper-quintile drugs at age 5 did not accumulate any study between ages 6 and 10 (2% in *Clinical* and 0% in *Literature*), which would be the equivalent of “slamming on the brakes.” Thus, the “life cycle of successful drugs” is different from that of genes studied in the literature [12], [13].

An example of a *Clinical* drug at the 87th percentile among those from the 1998 cohort is posaconazole, with 19 studies accumulated over the period 1998–2010. Posaconazole is an antifungal drug marketed under the brand name Noxafil. Five posaconazole *Clinical* studies were started in the more than 8 years before posaconazole was approved by the Food and Drug Administration (FDA) and the European Medicines Agency (EMEA) in 2006. Fourteen studies were started after FDA approval in the subsequent 4-year period between 2007 and 2010. Thus, regulatory approval probably led to acceleration in the growth of posaconazole studies.

Post-regulatory approval studies of posaconazole sought to broaden the types of infections and patient subpopulations amenable to posaconazole treatment. In fact, the developers of posaconazole had probably anticipated regulatory success and had increased the number of posaconazole studies even before approval had been granted. The cumulative number of studies for posaconazole went from being 3 at age 5 to being 11 at age 10. Thus, the rate of studies went from 0.6 per year during the first 5-year period to 1.6 per year during the second 5-year period. As has been mentioned, such acceleration in the rate of studies is, on average, a pattern of drugs meeting clinical success.

### Drug-centric disease taxonomy

Historically, diseases have been classified according to different criteria, such as pathology, anatomy and prognosis. More recently, new nosological criteria have been set forth based on molecular biology and genetics, such as disease genes, genetic associations and pathways [14–18]. Here, instead, I have explored the creation of a drug-centric disease taxonomy by classifying diseases according to the drugs they adopt.

**Fig 3:**
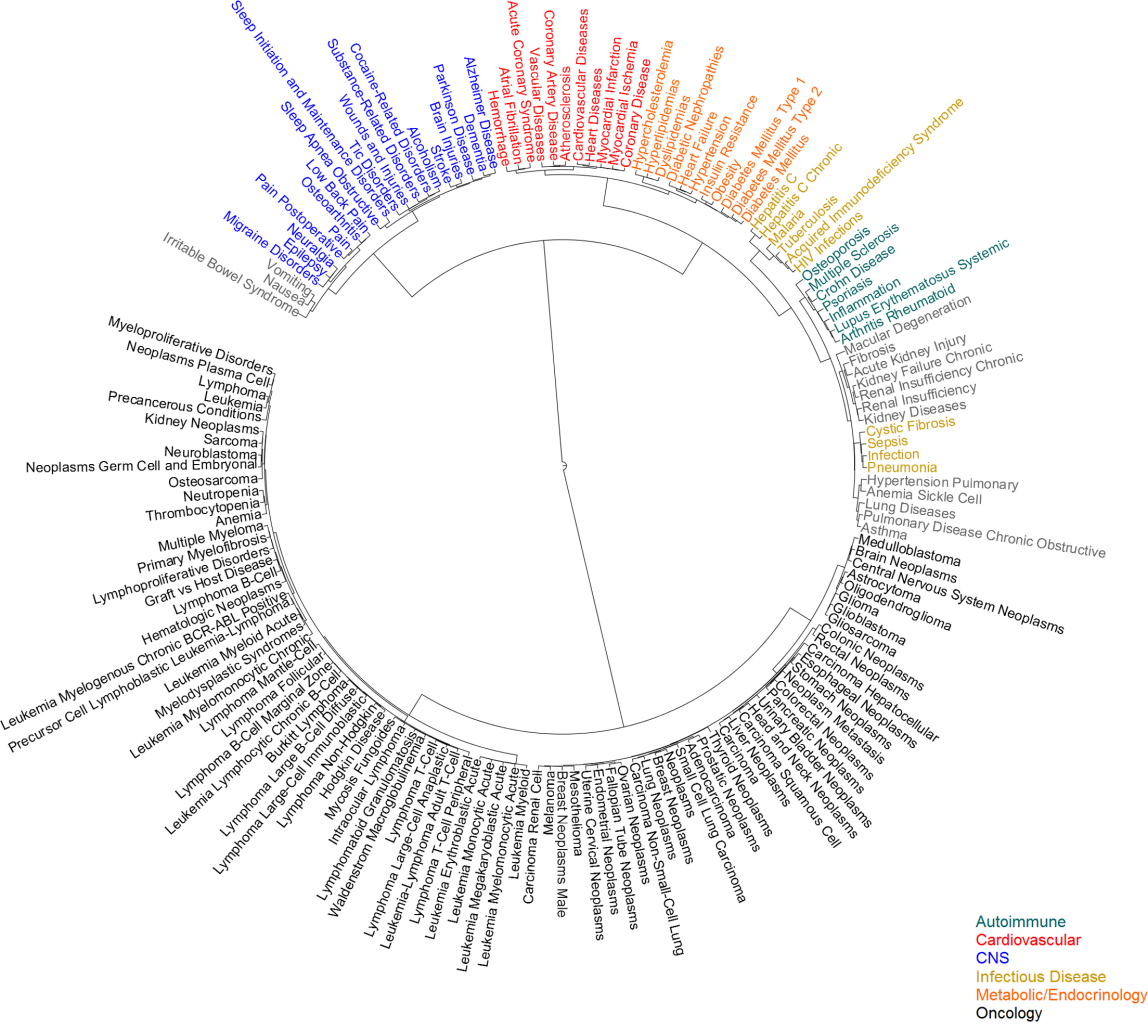
Drug-centric hierarchical clustering of diseases. One hundred fifty-six diseases from the *Clinical* dataset were clustered using a distance metric based on the drugs adopted by each disease. Several clusters were colored based on the class of diseases that prevailed in the cluster: autoimmune/inflammation (blue-green), cardiovascular (red), CNS (blue), infectious disease (gold), metabolic/endocrinology (orange), oncology (black).

Fig 3 shows an example of taxonomy built using hierarchical clustering based on the drugs adopted by diseases in the *Clinical* dataset. Only diseases that had adopted at least 50 drugs were selected for this analysis. Exploring this taxonomy illustrates some of the properties that a drug-centered disease taxonomy exhibits. For example, fundamental pathological similarities drive the creation of certain groupings. Cardio-metabolic diseases are clustered closely, mirroring the intertwined pathologies of “diabesity” and cardiovascular diseases. Organ- and tissue-based classifications arise for some diseases of the kidney, lung and CNS. Within the large oncology cluster, hematological cancers and CNS cancers tend to cluster together.

Some diseases in the drug-centric taxonomy are clustered with other diseases due to the fact that they are typical comorbidities or secondary diseases, or due to symptom similarity. Thus, neutropenia, thrombocytopenia and anemia appear within the oncology cluster. Fibrosis is grouped with renal diseases and macular degeneration, and vomiting and nausea are grouped with irritable bowel syndrome. Osteoporosis is classified within the autoimmune disease cluster probably because of the importance of inflammation in this disease, while sickle cell anemia appears with lung diseases possibly because pulmonary complications are common in sickle cell anemia.

Symptom similarity brings together in the taxonomy cystic fibrosis with infectious diseases, while osteoarthritis clusters with pain conditions, which reflects the scarcity of osteoarthritis drugs beyond those used for pain relief. Thus, some drugs may affect certain parts of the phenotype that is shared by several, otherwise fairly different diseases. For example graft-versus-host disease (GvHD) is classified within the oncology cluster due to the immunosuppressive effect of many oncological and GvHD drugs.

Within the diseases in Fig 3 those in the oncology cluster have a high level of drug sharing. These diseases have a median of 1.5% drugs in exclusivity (not shared with other diseases in the taxonomy). Diseases in the infectious and autoimmune disease clusters, on the other hand, have a median drug exclusivity of 13.5% and 13.4%, respectively, which points to the differentiation of the drugs used for these diseases. Some diseases with high level of drug exclusivity within the taxonomy, and that, in principle because of that could be harder to classify, are irritable bowel syndrome (37.5%), sleep initiation and maintenance disorders (34.0%), malaria (29.3%), Alzheimer’s disease (23.8%) and osteoporosis (22.9%).

### Drug propagation dynamics and network modeling

The adoption of a drug by a disease depends on the specific properties of the drug, especially the first time the drug is adopted—meaning when the drug is born. However, after a drug’s birth, the decision by other diseases to adopt the drug may also depend on the history of diseases that have adopted the drug in the past. Thus, diseases that adopt a drug earlier in time may influence the later adoption of the drug by other diseases. We can measure this influence by looking at similarities in patterns of drug adoption by diseases over time. For example, if disease A consistently adopts drugs before disease B, we may infer that disease A influences the drug adoptions made by disease B. Conversely, if disease B does not adopt drugs that have been already adopted by disease A, we may infer that disease A does not influence disease B. One needs to be careful, however, not to overlook diseases that may mediate the influence existing between pairs of diseases. For example, disease A may appear to be influencing disease B but that influence may be mediated by a disease C, which adopts drugs after disease A and before disease B. Thus, by looking at global adoption patterns we can make conjectures about disease-disease influences. Such influences might not be symmetrical: a particular disease may influence another disease more than vice versa, or, in extreme cases, the influence may exist in only one direction.

To illustrate this further, we can look at an example pair of diseases of some biological similarity, such as the diseases of the joint osteoarthritis (OA) and rheumatoid arthritis (RA). In our two datasets, more drugs were adopted by RA than by OA (*Literature*: 943 vs. 622; *Clinical*: 109 vs. 93). The *Literature* dataset indicates that drugs that have been adopted by both diseases are more likely to have been first adopted by RA than by OA (*Literature*: 401 vs. 103, p < 1 10^−15^) (in this section, p-values reported come from two-tailed t-tests). The *Clinical* dataset does not show such a tendency, perhaps due to its sparseness (8 vs. 13). Interestingly, however, the average time it takes for a drug to be adopted by OA after having been adopted by RA is larger than in the reverse direction (*Literature*: 14.6 ± 0.5 yrs vs. 7.7 ± 0.7 yrs, p < 2 10^−14^). Note that study dates are based on publication date in the *Literature* dataset and on trial start date in the *Clinical* dataset. Time differences can thus vary depending on study duration and time lag in publication of findings.

These results can be explained by delving deeper into the drugs adopted by both RA and OA. For example, a common RA drug with the brand name of Humira (adalimumab) was adopted by RA in the *Literature* dataset 4.8 yrs before it was adopted by OA. Meloxicam, an FDA-approved drug for OA, was adopted by OA in the *Literature* dataset 1.0 yrs before it was adopted by RA, while in the *Clinical* dataset the difference was 2.2 yrs. The difference in modes of action between Humira and meloxicam could justify such a sequence of events. Humira is a TNF-alpha inhibitor effective in a number of autoimmune diseases; therefore its adoption by RA is more biologically supported than by OA—hence, the delay in OA’s adoption. Meloxicam, on the other hand, is a non-steroidal anti-inflammatory drug (NSAID). NSAIDs are prescribed for both OA and RA, therefore any NSAID that showed promise in OA could be presumed to have a chance to work in RA, leading to a quicker adoption by RA. Thus, similarities and differences in disease biology and disease response to certain drug classes might explain some drug adoption patterns.

Such data, however, might not be conclusive enough to firmly establish the influence between diseases, even in cases in which this could be likely. An example is the pair of diseases psoriasis and atopic dermatitis, both diseases of the skin with an important pathological role by the immune system. In both datasets, more drugs had been first adopted by psoriasis, and later adopted by atopic dermatitis, than vice versa (*Literature*: 109 vs. 48; *Clinical*: 7 vs. 3). However, such numerical differences were only significant in the case of *Literature* (p < 2 10^−6^, p < 0.35, respectively).

In both datasets, the average delay for a drug to move from psoriasis to atopic dermatitis was smaller than in the reverse direction (*Literature*: 14.6 ± 0.5 yrs vs. 8.2 ± 0.6 yrs; *Clinical*: 5.3 ± 1.3 yrs vs. 2.9 ± 2.5 yrs; p < 4 10^−4^, p < 0.73, respectively) but only in the *Literature* case the difference was significant. Thus, while psoriasis and atopic dermatitis may influence each other’s drug adoptions, a pattern of one of the diseases being an “early adopter” for the other disease is not confirmed.

One can go from analyzing pairs of diseases to looking at disease families or the entire diseasome by attempting to build a network of relationships existing across multiple diseases on the basis of their drug adoption patterns. I developed one such network by comparing disease-disease influences to a communication network in which messages are sent following Poisson processes (see Methods). The messages in this network are the drugs that diseases “send” to each other. One advantage of such modeling is that we can estimate the average time it takes for a disease to send a drug to another disease.

**Fig 4:**
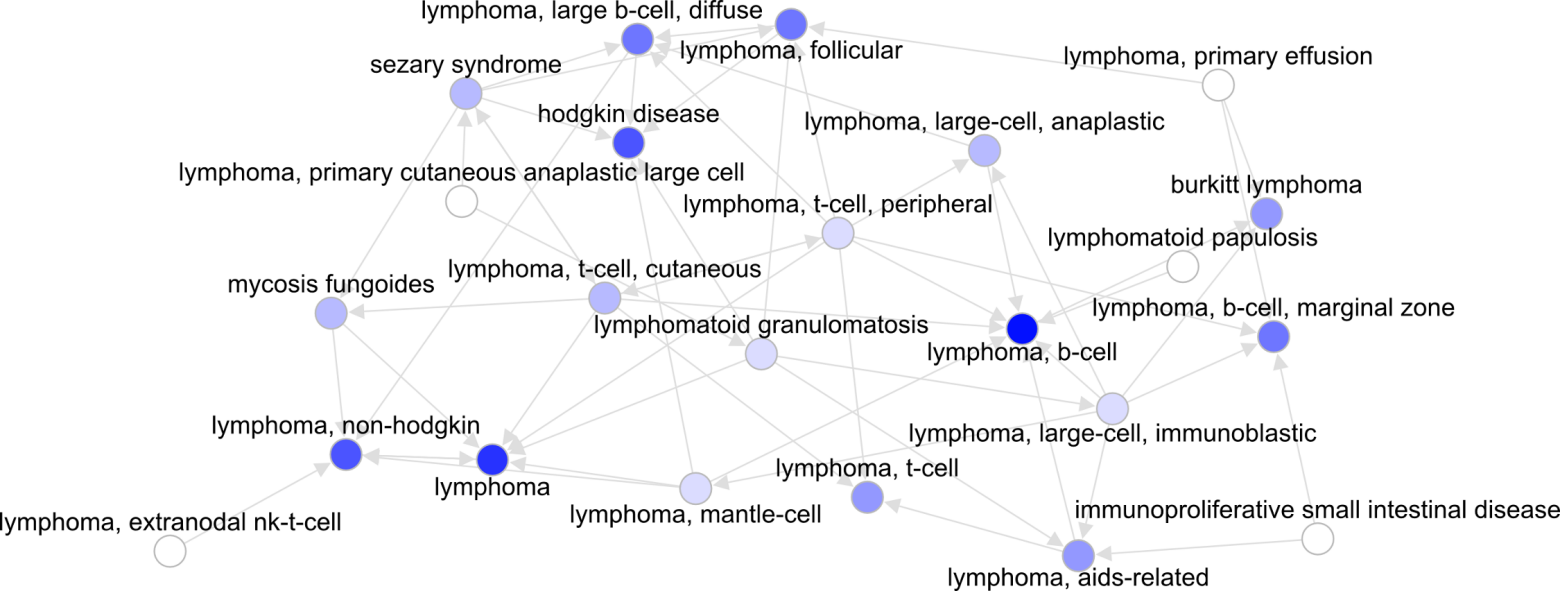
Lymphoma subnetwork based on the *Literature* dataset. Node colors are based on in-degree.

An example of subnetwork can be seen in Fig 4, comprising lymphoma cancers from the *Literature* dataset. In this network, diseases present out-going connections to other diseases (in network topology the sum of these connections is called the “out-degree”) and receive connections from other diseases (in-degree). The most connected diseases within this subnetwork are T-cell peripheral lymphoma, with 9 connections, and B-cell lymphoma and T-cell cutaneous lymphoma, with 8 connections each. T-cell peripheral lymphoma (out-degree = 8, in-degree = 1) and T-cell cutaneous lymphoma (out-degree = 6, in-degree = 2) are more typically a source of connections while B-cell lymphoma is more typically a destination of connections (out-degree = 1, in-degree = 7). Reflecting this dynamic, the common chemotherapeutic cyclophosphamide was adopted in the *Literature* dataset by a number of lymphomas such as Hodgkin disease, mycosis fungoides and non-Hodgkin lymphoma earlier than by B-cell lymphoma. The most disconnected diseases are lymphomatoid papulosis and extranodal NK-T-cell lymphoma (both with out-degree = 1). Lymphomatoid papulosis is a disease difficult to classify and its relation to lymphomas is weak. Extranodal NK-T-cell lymphoma is connected to non-Hodgkin lymphoma, of which it is a subtype. In terms of network centrality, the disease with the shortest median path to the rest of diseases is primary cutaneous anaplastic large cell lymphoma. The diseases to which other diseases have the median shortest path are non-Hodgkin lymphoma and Hodgkin’s disease, which are the most common lymphomas.

### Further validation

**Fig 5:**
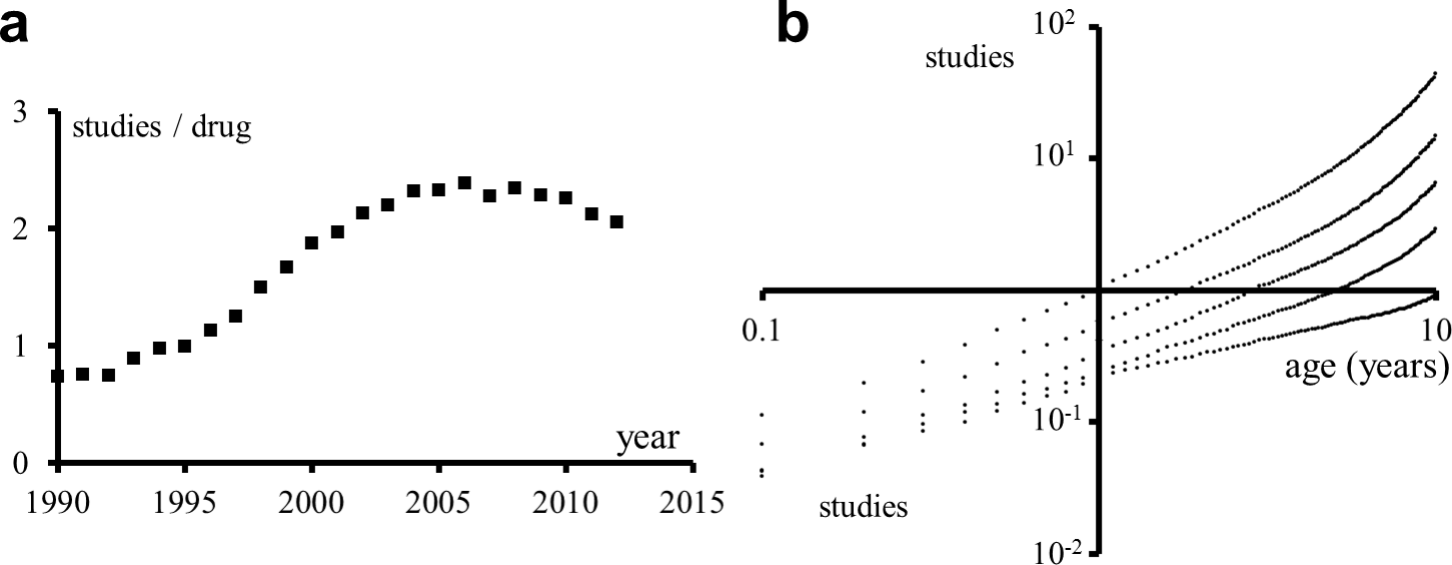
Average number of studies per drug per year and average cumulative number of studies for drugs born during the period 1990–2010. (a) Average number of studies per drug per year in the *Trial* dataset. (b) Average cumulative number of studies for drugs born during the period 1990–2010 in the *Trial* dataset. The abscissa represents the age of the drug in years. Each of the five trends corresponds to a quintile of the dataset, with the most studied drugs at the top and the least studied at the bottom (excluding the first study). As would be expected, the value of *β* increases for each quintile. Regression curves for every trend are power laws with, respectively, the following *β* and *R*^*2*^ values, from upper quintile to lower quintile: *β* = {1.39, 1.22, 1.13, 0.94, 0.64} and *R*^*2*^ = {0.97, 0.97, 0.98, 0.98, 0.99}.

To further validate the results described, several analyses were repeated using a proprietary clinical trial dataset from the company Citeline containing manually normalized drug and disease names (*Trial* dataset). Fig 5 shows analyses with the *Trial* dataset analogous to those in Fig 1 and Fig 2. It can be observed that the trends are similar to those corresponding to the *Clinical* and *Literature* datasets. Thus, automatic annotation does not produce a noticeable bias in the results over manual annotation.

**Fig 6:**
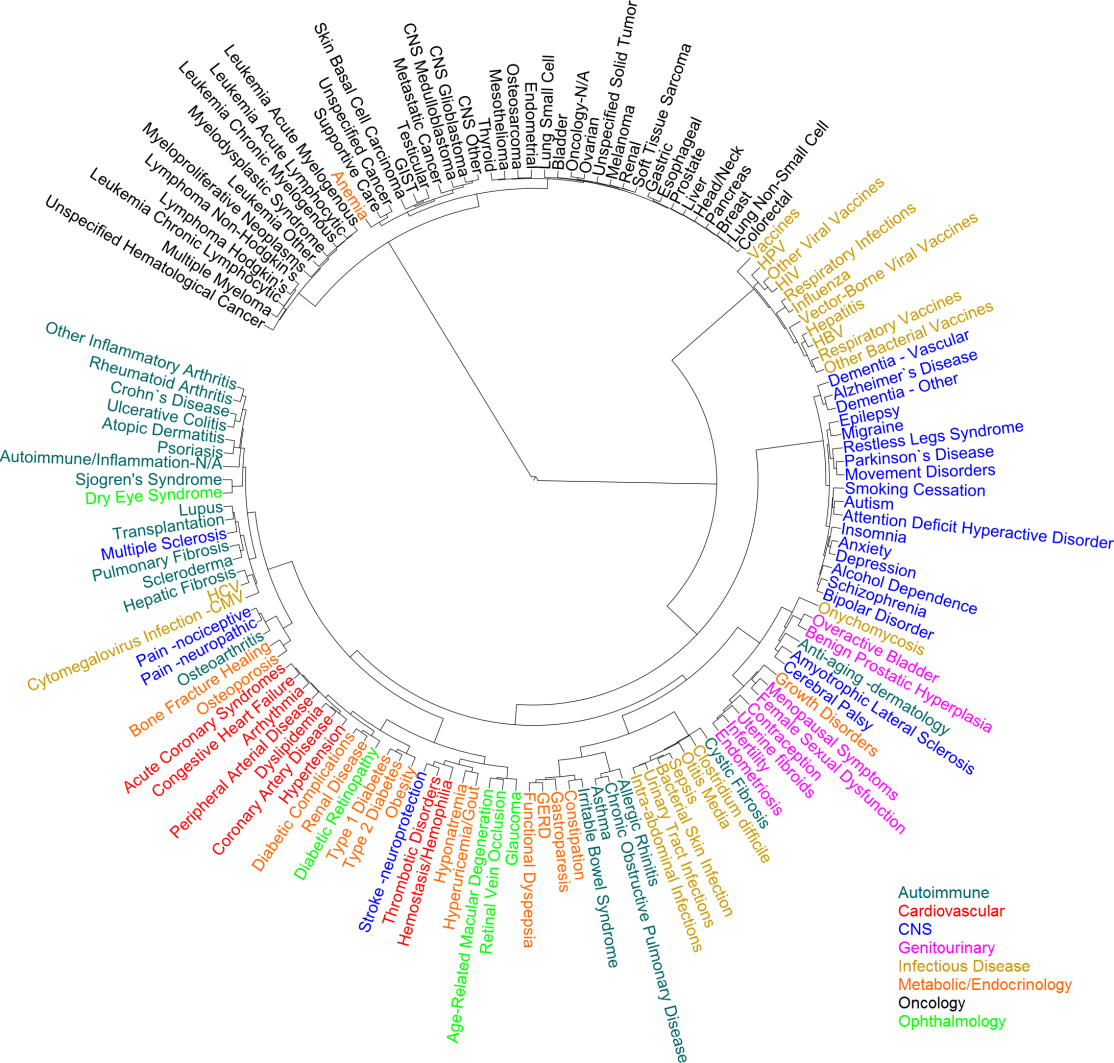
Drug-centric hierarchical clustering of diseases. One hundred forty-one diseases from the *Trial* dataset were clustered using a distance metric based on the drugs adopted by each disease. The *Trial* manually-created classification included the categories: autoimmune/inflammation (blue-green), cardiovascular (red), CNS (blue), genitourinary (pink), infectious disease (gold), metabolic/endocrinology (orange), oncology (black), ophthalmology (green).

Fig 6 shows a taxonomy built using hierarchical clustering based on the drugs adopted by each disease in the *Trial* dataset. Many of the grouping tendencies are similar to those in the *Clinical* taxonomy already shown. Additionally, the *Trial* dataset includes a manually-created disease classification that can be compared with the clustering algorithm-created taxonomy. For example, some disease groupings in the drug-centric taxonomy that differ from those in the manually-created *Trial* taxonomy reflect different views of the underlying disease biology. Thus, multiple sclerosis is grouped in the algorithm-created taxonomy with autoimmune/inflammation diseases rather than with CNS diseases, based on the fact that multiple sclerosis drugs mainly target the immune system. Irritable bowel syndrome is grouped with metabolic/endocrinology diseases rather than with autoimmune/inflammation diseases, which fits better the biology of IBS. Stroke is grouped with cardiovascular diseases rather than with CNS diseases, due to its relation with cardiovascular diseases such as thrombotic disorders.

The cardio-metabolic spectrum emerges in the drug-centric taxonomy in Fig 6 with the grouping of diabetes type 1 and diabetes type 2 with diabetic complications, renal diseases, diabetic retinopathy and obesity. These, in turn, appear closely associated with cardiovascular diseases such as acute coronary syndrome and dyslipidemia.

Symptom similarity forms the basis for some disease groupings, such as that of osteoarthritis with neuropathic and nociceptive pain. Other such examples are the groupings of cystic fibrosis, which is characterized by chronic infections, with infectious diseases; and of dry eye syndrome with Sjögren's syndrome, of which dry eyes is a major symptom.

Certain disease classifications are more organ- and tissue-based such as, for example, the lung-related group of asthma, chronic obstructive pulmonary disease (COPD) and allergic rhinitis. In oncology, blood cancers and CNS cancers appear as two distinguishable groups. The remaining cancer subtypes, however, do not appear to be grouped following clear patterns, which may point to the non-specificity of a number of cancer drugs.

Other diseases are classified based on their being a consequence or a co-morbidity of another disease, such as anemia with oncology; hepatic fibrosis with both cytomegalovirus (CMV) infection and hepatitis C virus (HCV); and migraine with epilepsy.

Finally, some diseases do not seem to cluster with other diseases. Examples are the pair of diseases amyotrophic lateral sclerosis and cerebral palsy, and the pair of diseases overactive bladder and benign prostatic hyperplasia. Individual diseases such as the fungal infection onychomycosis, growth disorders, and anti-aging also form their own distinct clusters. Interestingly, these are diseases with a large percentage of drugs that are unique to them and not shared with any other disease in the taxonomy. Onychomycosis and anti-aging are first and second among diseases in the uniqueness of their drugs, with 80% and 77%, respectively, being exclusive to them. Growth disorders has 50% of its drugs in exclusivity, compared to a median exclusivity of 15% among all diseases in the dataset. By comparison, the median oncological disease has a 6% drug exclusivity. Thus, the structure of drug-centric taxonomies depends on the stage of development, differentiation and exclusivity of existing therapies. It is worth noting also the different overall levels of drug exclusivity found in the *Trial* dataset vs. the *Clinical* dataset, reflecting the differences in granularity of the manual and automatic annotations.

**Fig 7:**
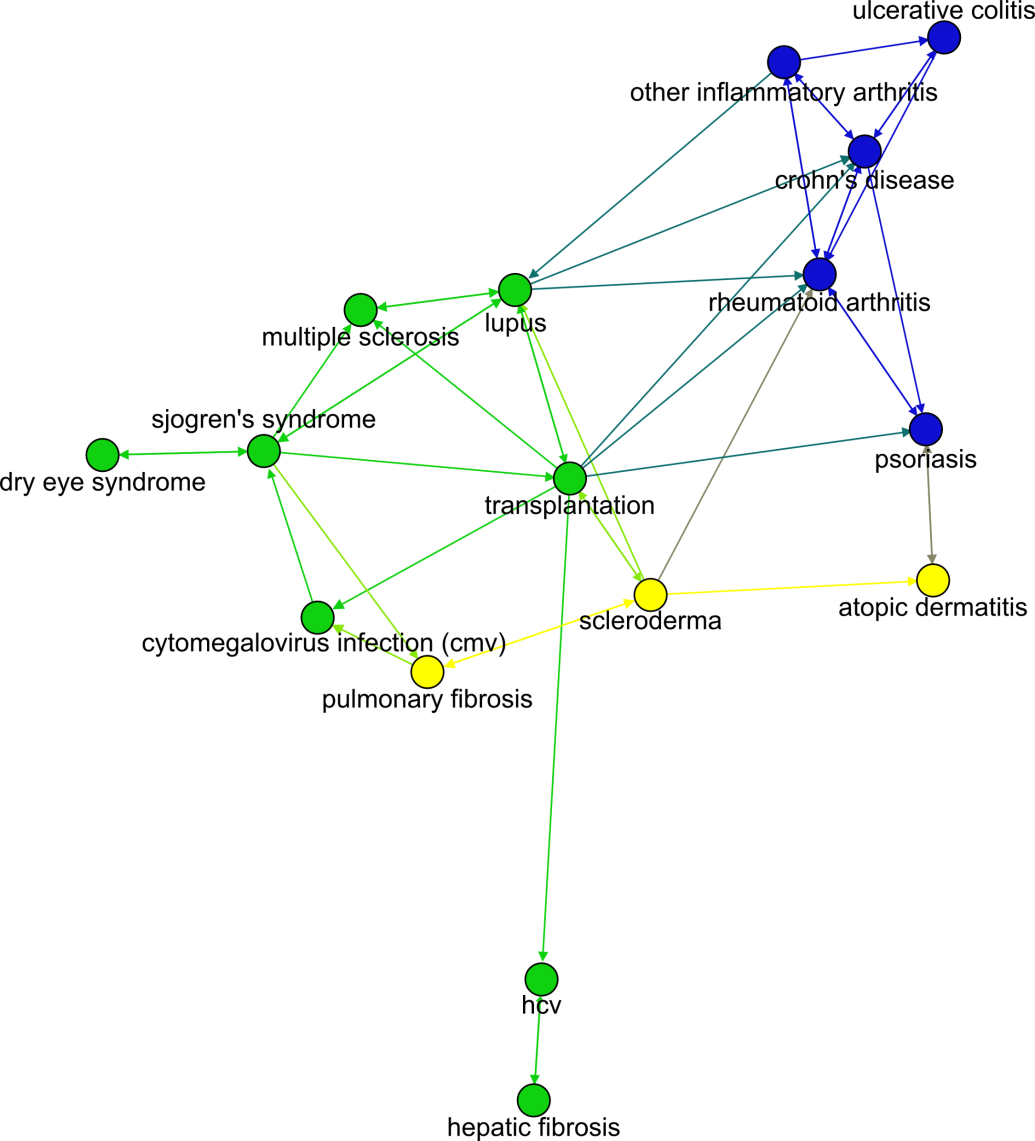
Autoimmune subnetwork based on the *Trial* dataset. Node colors reflect modularity.

In Fig 7, a section of a network based on the *Trial* dataset focused on the autoimmune disease cluster (from Fig 6) can be seen. As could be expected, diseases within the autoimmune cluster are highly connected with each other. The diseases with most connections are transplantation, which is connected to 9 other diseases, and rheumatoid arthritis and lupus, which are connected to 7. Interestingly, transplantation is more typically a source than a destination of connections (out-degree = 8, in-degree = 3) while rheumatoid arthritis is more typically a destination of connections (out-degree = 3, in-degree = 7). Therefore, it would seem that transplantation is an “early adopter” disease for drugs entering this cluster of autoimmune diseases while rheumatoid arthritis is the disease most diversely influenced within the cluster. Transplantation can plausibly be an early adopter for immunological diseases as transplantation drugs are generally immunosuppressive and thus can have therapeutic potential in other autoimmune diseases. An example of such drug in the *Trial* dataset is the immune suppressor cyclosporine, which was born as an application for transplantation and then widely applied in other immunological diseases. Transplantation, in fact, exhibits a higher out-degree (n = 17) than in-degree (n = 6) when the entire *Trial* disease network is considered. (For comparison, both the median in-degree and out-degree in the network are 6.) The disease to which other diseases have the median shortest path in the autoimmune cluster, on the other hand, is rheumatoid arthritis. Four diseases exhibit the shortest average paths to the rest of diseases in the cluster: lupus, scleroderma, Sjögren’s disease and transplantation. HCV and hepatic fibrosis are the most detached diseases of the cluster.

## Discussion

Here it has been shown that the total number of studies involving drugs have been increasing faster that the number of unique drugs under study. Interestingly, it has been noted elsewhere that, despite the increase in importance of target-based drug discovery, the diversity of small molecules studied for diseases has been growing much slower, on average, than the diversity of genes [19]. This may suggest that, while the chemical and biological space that can be explored with current drug development technologies is theoretically vast [20–22], the set of therapeutically or biologically relevant molecules might be far more restricted [23], [24]. Thus, the intrinsic value of drugs derives in part from their relative scarcity. Anyone trying to develop a drug against an established disease target will often find that other drug developers have converged to (and patented) similar solutions. It is also interesting to note that “follow-on” drugs that target the same mechanism as other existing drugs are not as common as believed [25].

Additionally, I have shown that successful drugs accelerate their accumulation of studies, following a power law, and that this accumulation is correlated with clinical phase advancement. Successful drugs become “rich” in number of studies, and are the subject of a large percentage of all studies. This success has momentum, so that the accumulation of studies over a period of time is likely to continue over the next period of time. Such dynamics could suggest a feedback loop in which drugs that initially attract attention randomly end up accumulating more studies than other drugs in a “rich get richer” fashion. This pattern has been proposed for publications about genes [13]. However, the outcomes of the studies analyzed here can be judged with rather objective measures such as clinical advancement and thus it is hard to argue for multiplicative noise effects unrelated to clinical results. Moreover, for most drugs success is time-limited and followed by stagnation or decay in the rate of accumulation rather than continued accelerating growth.

This study has also shown that drugs can define relationships between diseases. One type of relationship is that embodied by the drug-centric disease taxonomy explored here. Such taxonomy, however, does not take into account the timing of drug adoptions. Thus, I created a network model in which connections reflect drug adoption dynamics. The network connectivity is associated to drug propagation delays and represents the degree of influence that diseases have over each other. This network portrays drugs as technologies that can propagate from disease to disease following diffusion of innovation patterns [26]. Some diseases might be quick at testing new drugs and be described as “innovators” or “early adopters,” while other diseases might be slow or “late adopters.” From the point of view of a drug developer, a disease may be approached first as an “early adopter” because it represents a lower hurdle due to reduced cost and length of clinical trials or a less challenging competitive landscape [27]. The variability and asymmetry of drug adoption delays hint that there could be opportunities for drug developers to monitor more effectively drugs in diseases connected to their diseases of focus or to expand the testing of their drugs into diseases ahead of competitors.

The networks I presented were created under a number of assumptions. In particular, I considered that diseases communicate with each other following a Poisson process with a fixed rate. Or, in other words, that pairs of diseases communicate at a constant average rate and independently of past history. This simplification obviates that the rate of communication between diseases may change over time, due to, for example, an evolving understanding of the biology of the diseases. Moreover, the modeling presented does not take into account that drug subsets can be communicated at different rates between pairs of diseases. For example, angiogenesis inhibitors are drugs well suited for many oncological diseases, such as colon cancer, as well as for other non-oncological diseases, such as wet age-related macular degeneration (AMD). However, due to the biological differences that exist between wet AMD and colon cancer, many other colon cancer drugs may never be adopted by wet AMD.

The network modelling also neglected any event not described by drug adoption times that could be mediating drug adoption patterns. Perhaps most saliently, it did not take into account the importance that a drug may have for a disease. Certainly a drug that has been heavily studied within the context of a disease, that has achieved regulatory approvals, or that has been described in high impact publications, should have a greater potential to be adopted by other diseases. For example, it has been shown that there is a correlation between the number of studies that exist for a drug and the average impact factor of the journals in which the studies are published [28]. Thus, a thoroughly-studied drug has more visibility and, therefore, more potential to be adopted by diseases. The model here presented, however, focuses purely on drug adoption times by diseases rather than how often they are studied or in which journals the studies are published. A potential improvement of the model could be to modify the probability of a drug being sent from one disease to another based on the drug’s importance for the sending disease.

It is crucial to stress that a drug’s relationship to a disease can take many forms. Some drugs can be efficacious and safe against a disease but not as efficacious and safe as existing standards of care and therefore be a “neglected drug.” Drugs believed to have a therapeutic effect can turn out to be no better than placebo, as with some antidepressant drugs [29], and results from clinical trials can be statistically misinterpreted [30]. Some drugs might be effective only in small patient subpopulations. In short, the relationships between drugs and diseases are complex and regulatory approval is only one standard of measurement. Thus, the “eurekometrics” [31] of pairing diseases and drugs are not rigid. Ideally, for example, the analysis would also have involved drug off-label use. Chiang & Butte [32], for example, utilized the proprietary DRUGDEX database as source of off-label drug use information, and Jung et al. [33] identified off-label drug use in the STRIDE database of medical records. The analysis could also have taken into account finer-grained disease subtypes and patient subpopulations.

Drugs are often seen as tools in a pharmacological endeavor driven by biological and medical practice. However, drugs interact with diseases not just through their therapeutic value but also by helping to elucidate the biology of diseases. As Rein Voss described in his work *Drugs looking for diseases* [34], advances in pharmacological therapy are based not only on increased knowledge about the nature of diseases but also on increased understanding of the principles of drug action. Thus, research on diseases and drugs reinforce each other.

## Methods

Three datasets were used in the analysis, which were called respectively *Literature*, *Clinical* and *Trial*. The *Literature* dataset comprised 2 598 877 Medline records annotated with drug names from PharmGKB and DrugBank by the GeneView text mining tool ([35], download 08-Aug-2014) and annotated with “major topic” Medical Subject Heading (MeSH) terms corresponding to the branch Diseases (branch C) of the MeSH tree structure. The *Clinical* dataset concerned 91 796 studies from the website ClinicalTrials.gov that mention a “Drug” intervention type, an indication and a trial start date—out of the 172 262 studies present in the website ClinicalTrials.gov (download 05-Aug-2014). Drug names were mapped to standard identifiers using the STITCH aliases for chemicals ([36], v4, download 09-Aug-2015). Indication names were mapped to MeSH terms using synonyms from the MeSH Diseases branch. Of the 91 796 studies from ClinicalTrials.gov under consideration, 47 205 were mapped to at least one drug in STITCH and one disease in MeSH. The *Trial* dataset spanned 169 345 clinical trial descriptions up to 2013 collected by the company Citeline. These descriptions include normalized drug names, normalized disease names and a therapeutic area assignment for each disease. The *Trial* dataset was included in the study to validate the results from the analysis of the automatically-normalized *Clinical* and *Literature* datasets. Both *Literature* and *Trial* use the denomination “disease” to describe the condition aimed at by a drug study. However, *Clinical* uses instead the denomination “condition.” To simplify, I have used “disease” throughout this study, even in cases where a drug is not being applied against a disease, such as in the case of transplantation.

Each record in each of the three datasets is called here a “study.” For each of the datasets, I defined ***d*** *= {d*_*k*_*}* as the set of all drugs that have been used to treat the set of all diseases ***D*** *= {D*_*i*_*}* over all the studies ***s*** *= {s*_*l*_*}* in the dataset. For *Literature*, the number of unique drugs was |***d***| = 2 452 and the number of unique diseases |***D***| = 4 522. For *Trial*, |***d***| = 25 982 and |***D***| = 149. For *Clinical*, |***d***| = 3 572 and |***D***| = 1 865.

For every drug *d*_*k*_ mentioned in a study *s*_*l*_, I created a triplet $\left\{d_{k},s_{l},t_{s_{l}}^{d_{k}}\right\}$, in which $t_{s_{l}}^{d_{k}}$ is a timestamp corresponding to either the starting date of the clinical trial in the *Trial* and *Clinical* datasets, or the publication date in the *Literature* dataset. The average number of studies per drug over a period of time *(t*_*0*_, *t*_*1*_*)* is the ratio

$$\frac{|\left\{d_{k}|\forall s_{l},\exists t_{0}<t_{s_{l}}^{d_{k}}<t_{1}\}\right|}{\left|\left\{s_{l}|\forall d_{k},{\exists t}_{0}<t_{s_{l}}^{d_{k}}<t_{1}\right\}\right|}.$$ (1)

The cumulative number of studies for a drug *d*_*k*_ over time is the function

$$F_{d_{k}}\left(t\right)=\left|\left\{s_{l}|t_{s_{l}}^{d_{k}}\leq t\right\}\right|.$$ (2)

The derivative ${F^{\prime }}_{d_{k}}\left(t\right)$ of this function is the rate of studies per drug at time *t*. To compare the evolution of $F_{d_{k}}\left(t\right)$ for different drugs *d*_*k*_ I aligned them by subtracting the time $t_{min}^{d_{k}}\in \left\{t_{s_{l}}^{d_{k}}\right\}$ in which a drug is studied for the first time

$${\forall s_{l},t}_{min}^{d_{k}}\leq t_{s_{l}}^{d_{k}}$$ (3)

so that

$$F_{d_{k}}\left(t\prime \right)=\left|\left\{s_{l}|t_{s_{l}}^{d_{k}}-t_{min}^{d_{k}}\leq t\prime \right\}\right|,$$ (4)

where $0\leq t^{\prime }\leq t_{max}-t_{min}^{d_{k}}$ and *t*_*max*_ is the last observation time (here, the end of year 2010). The time $t_{min}^{d_{k}}$ is called here the birth time of drug *d*_*k*_. The average cumulative function is then

$$F\left(t^{\prime }\right)=\frac{1}{\left|\left\{d_{k}|t\prime \leq t_{max}-t_{min}^{d_{k}}\right\}\right|}\sum_{\left|\left\{d_{k}|t^{\prime }\leq t_{max}-t_{min}^{d_{k}}\right\}\right|}F_{d_{k}}\left(t^{\prime }\right).$$ (5)

Notice that *F*(*t*′) is not necessarily a monotonically increasing function as each $F_{d_{k}}\left(t\prime \right)$ covers a different time span $\left[0,t_{max}-t_{min}^{d_{k}}\right]$. For the calculation of *F*(*t*′) I excluded for each drug the first study, as it would only have shifted the function up by one. Drugs for which there was only one study available were excluded. Using the median of all $\left\{F_{d_{k}}\left(t\prime \right)\right\}$ for every time *t* instead of the average function *F(t’)* yields analogous results.

In each dataset, a disease *D*_*i*_ *∈* ***D*** adopts a drug *d*_*k*_ *∈* ***d*** the first time that drug is studied together with the disease, with $t_{D_{i}}^{d_{k}}$ being the time of that event. Thus, a drug adoption by a disease is defined by the triplet $\left\{d_{k},D_{i},t_{D_{i}}^{d_{k}}\right\}$. The cumulative number of diseases that adopt a drug *d*_*k*_ is defined by

$$G_{d_{k}}\left(t\right)=\left|\left\{D_{i}|t_{D_{i}}^{d_{k}}\leq t\right\}\right|.$$ (6)

The derivative ${G^{\prime }}_{d_{k}}\left(t\right)$ of this cumulative function is the rate of diseases that adopt a drug *d*_*k*_ at time *t*. Similarly as with $F_{d_{k}}(t)$, in order to compare the evolution of $G_{d_{k}}(t)$ for different drugs *d*_*k*_, I time-shifted them by subtracting the time $t_{min}^{d_{k}}$ in which a drug was adopted for the first time by a disease, so that:

$$G_{d_{k}}\left(t\prime \right)=\left|\left\{D_{i}|t_{D_{i}}^{d_{k}}-t_{min}^{d_{k}}\leq t\prime \right\}\right|,$$ (7)

where $0\leq t\prime \leq t_{max}-t_{min}^{d_{k}}.$. The average cumulative function is then

$$G\left(t\prime \right)=\frac{1}{\left|\left\{d_{k}|t\prime \leq t_{max}-t_{min}^{d_{k}}\right\}\right|}\sum_{\left|\left\{d_{k}|t^{\prime }\leq t_{max}-t_{min}^{d_{k}}\right\}\right|}G_{d_{k}}\left(t\prime \right).$$ (8)

Notice that *G*(*t*′) is not necessarily a monotonically increasing function. For the calculation of *G*(*t*′), I excluded for each drug the first disease that adopted the drug. Drugs that were adopted by only one disease were excluded as well.

To group drugs according to the number of studies they have accumulated, I looked at all drugs whose time of birth $t_{min}^{d_{k}}$ was within a certain year and then separated them into groups according to the cumulative number of adoptions they had reached by *t*_*max*_, $G_{d_{k}}\left({t=t}_{max}\right)$, or after a certain time interval *Δt*. Thus, drugs were not penalized or favored by comparing them to drugs born in earlier or later years. Similarly, drugs were also grouped based on the values of $F_{d_{k}}\left({t=t}_{max}\right)$ and $F_{d_{k}}\left(\Delta t\right)$.

To create disease taxonomies I used hierarchical clustering employing Ward's minimum variance method. As metric for the clustering, I used a measure of mutual information between diseases based on the number of drugs they had adopted in common. The general definition of mutual information is:

$$I(x;y)=\sum_{x,y}p\left(x,y\right)\text{log}\;\left(\frac{p(x,y)}{p\left(x\right)p\left(y\right)}\right).$$ (9)

In this case, the *p(x*,*y)* function is a two-dimensional Bernoulli distribution. Given that |***d***| is the number of all drugs that have been adopted by all diseases, the *p(x*,*y)* for a pair of diseases *D*_*i*_ and *D*_*j*_ is defined as

$$p^{ij}\left(x,y\right)=\frac{1}{\left|d\right|}\left[\begin{array}{cc} \left|d-\left(d^{i}\cup d^{j}\right)\right| & \left|d^{i}-d^{j}\right|\\ \left|d^{j}-d^{i}\right| & \left|d^{i}\cap d^{j}\right| \end{array}\right],$$ (10)

where ***d***^*i*^ *⊂* ***d*** is the set of drugs that has been adopted by disease *D*_*i*_ *∈ D*. The mutual information metric used for the clustering was then

$$I\left(D_{i};D_{j}\right)=\sum_{\begin{array}{c} y=\left[0,1\right]\\ x=\left[0,1\right] \end{array}}p^{ij}\left(x,y\right)\text{log}\;\left(\frac{p^{ij}\left(x,y\right)}{p^{ij}\left(x\right)p^{ij}\left(y\right)}\right).$$ (11)

I considered a drug exclusive to a disease if only one disease had adopted it within the group of drugs in consideration. Thus, a drug *d*_*k*_ adopted by disease *D*_*i*_ (*d*_*k*_ *∈* ***d***^*i*^) is exclusive to disease *D*_*i*_ with respect to all diseases ***d***, if ∀*j* ≠ *i*, *d*_*k*_ ∉ ***d***^*j*^.

When a drug is adopted by a disease, there can be a change in the probability that the drug will be adopted by other, similar diseases. This process was modelled as a communication network in which diseases send drugs to each other. A disease *D*_*i*_ can only send a drug *d*_*k*_ to another disease *D*_*j*_, if the drug *d*_*k*_ has been adopted by disease *D*_*i*_ but has not been adopted yet by disease *D*_*j*_. Such a communication network can be described by a directed graph in which each node is a disease. Every edge of the network has a message propagation delay associated, which is the average time that a disease takes to send a drug to another disease.

The goal was to reverse-engineer this communication network knowing only drug adoption times by diseases. For that, I employed Poisson processes, which are commonly used to model communication networks. The first approximation involved looking solely at pairs of diseases and not at the full network. Following the definition of a Poisson process, it is established that, for any drug *d*_*k*_ adopted by a disease *D*_*i*_ at time $t_{D_{i}}^{d_{k}}$, the probability of that drug being adopted by a disease *D*_*j*_ that has not yet adopted the drug depends only on the time between drug adoptions,

$$\Delta t_{D_{i}D_{j}}^{d_{k}}=t_{D_{j}}^{d_{k}}-t_{D_{i}}^{d_{k}},$$ (12)

such that

$$p\left(\Delta t_{D_{i}D_{j}}^{d_{k}}>t\right)=e^{-\lambda_{D_{i}D_{j}}t},$$ (13)

in which $t_{D_{j}}^{d_{k}}>t_{D_{i}}^{d_{k}}$. The subset of drugs covered by this definition is

$$d^{ij}=\left\{d_{k}\in d^{i}\cap d^{j}|t_{D_{j}}^{d_{k}}>t_{D_{i}}^{d_{k}}\right\}.$$ (14)

The value of $\lambda_{D_{i}D_{j}}$ in the Poisson process is informative because it is the average frequency of communication between diseases *D*_*i*_ and *D*_*j*_ and, hence, the inverse of the average propagation delay:

$$T_{D_{i}D_{j}}=\frac{1}{\lambda_{D_{i}D_{j}}}.$$ (15)

Thus, high values of $\lambda_{D_{i}D_{j}}$ represent low propagation delays while low values of $\lambda_{D_{i}D_{j}}$ represent large propagation delays. Since the nodes of the network are “competing” to be the first to pass messages to other nodes, propagation delays are an important factor to evaluate the level of influence that nodes have over each other.

To compute the value of each $\lambda_{D_{i}D_{j}}$ for each pair of diseases *D*_*i*_ and *D*_*j*_, I used the maximum likelihood estimate (MLE) [37] method using as sample data the $\Delta t_{D_{i}D_{j}}^{d_{k}}$ values observed for every drug *d*_*k*_ ∈ ***d***^*ij*^. The MLE likelihood function had this form in this case:

$$L(\lambda_{D_{i}D_{j}})\propto \prod_{d_{k}\in d^{ij}}p\;\left({\Delta t}_{D_{i}D_{j}}^{d_{k}}=t_{D_{j}}^{d_{k}}-t_{D_{i}}^{d_{k}}|\lambda_{D_{i}D_{j}}\right).$$ (16)

Thus, maximizing the value of the likelihood functions $L(\lambda_{D_{i}D_{j}})$ involved finding the set of $\left\{\lambda_{D_{i}D_{j}}\right\}$ that make most likely the observed drug adoption patterns. I also considered the cases in which a drug had been adopted by a disease *D*_*i*_ but not by a disease *D*_*j*_, i.e., *d*_*k*_ ∈ ***d***^*i*^ − ***d***^*j*^. The likelihood function in such cases was based on

$$L(\lambda_{D_{i}D_{j}})\propto \prod_{d_{k}\in d^{i}-d^{j}}p\left({\Delta t}_{D_{i}D_{j}}^{d_{k}}>t_{max}-t_{D_{i}}^{d_{k}}|\lambda_{D_{i}D_{j}}\right)$$ (17)

where *t*_*max*_ is the last observation time (here, the end of year 2010). Including such cases in the computation increases the number of data samples and penalizes connections between diseases in which one disease adopts a high number of drugs that are not adopted by another disease. Not considering this factor would lead diseases that have adopted a large number of drugs to unrealistically dominate the network. Thus, with this additional factor the MLE likelihood function looked like:

$$L\left(\lambda_{D_{i}D_{j}}\right)\propto \prod_{d_{k}\in d^{ij}}p\left({\Delta t}_{D_{i}D_{j}}^{d_{k}}=t_{D_{j}}^{d_{k}}-t_{D_{i}}^{d_{k}}|\lambda_{D_{i}D_{j}}\right)\prod_{d_{k}\in d^{i}-d^{j}}p\left({\Delta t}_{D_{i}D_{j}}^{d_{k}}>t_{max}-t_{D_{i}}^{d_{k}}|\lambda_{D_{i}D_{j}}\right)$$ (18)

which becomes

$$L\left(\lambda_{D_{i}D_{j}}\right)\propto \prod_{d_{k}\in d^{ij}}{\lambda_{D_{i}D_{j}}e}^{-\lambda_{D_{i}D_{j}}\left(t_{D_{j}}^{d_{k}}-t_{D_{i}}^{d_{k}}\right)}\prod_{d_{k}\in d^{i}-d^{j}}e^{-\lambda_{D_{i}D_{j}}\left(t_{max}-t_{D_{i}}^{d_{k}}\right)}.$$ (19)

To find the $\lambda_{D_{i}D_{j}}$ that maximizes $L\left(\lambda_{D_{i}D_{j}}\right)$, we can solve the equation

$$\frac{\partial L\left(\lambda_{D_{i}D_{j}}\right)}{\partial \lambda_{D_{i}D_{j}}}=0.$$ (20)

So far I have described a pair-wise model. A fuller picture can be built with a more detailed model that considers relations between all diseases. This model takes into account all drug adoptions of a drug *d*_*k*_ that happen before a disease *D*_*j*_ adopts the drug *d*_*k*_. Thus, every $\lambda_{D_{i}D_{j}}$ for a given disease *D*_*j*_ can be computed together by maximizing the function

$$L\left({\left\{\lambda_{D_{i}D_{j}}\right\}}_{\forall D_{i}}\right)=\prod_{\forall D_{i}}L\left(\lambda_{D_{i}D_{j}}\right),$$ (21)

using the equation

$$\frac{\partial L\left({\{\lambda_{D_{i}D_{j}}\}}_{\forall D_{i}}\right)}{\partial {\left\{\lambda_{D_{i}D_{j}}\right\}}_{\forall D_{i}}}=0.$$ (22)

This is easier to solve if we consider the log-likelihood function rather than the likelihood function:

$$\frac{\partial \text{log}\left(L\left({\left\{\lambda_{D_{i}D_{j}}\right\}}_{\forall D_{i}}\right)\right)}{{\left\{\partial \lambda_{D_{i}D_{j}}\right\}}_{\forall D_{i}}}=0.$$ (23)

After derivation, this equation can be solved numerically using a gradient descent method. This process can then be repeated for each disease *D*_*j*_. The networks presented in Fig 4 and Fig 7 show connections only between diseases with $T_{D_{i}D_{j}}=\frac{1}{\lambda_{D_{i}D_{j}}}<100$ yrs. For a $T_{D_{i}D_{j}}\geq 100$ yrs, the probability of a disease sending a drug to another disease within a 20-year period is lower than 19%. Of note is that data sparseness can be a limitation for the model described. For example, for diseases with a small number of drug adoptions, estimates for $\lambda_{D_{i}D_{j}}$ can be unrealistically high or low. Overall delay paths in disease networks were computed with Dijkstra’s algorithm using the $T_{D_{i}D_{j}}$ values as network distance metric.

In the text, time differences in the adoptions of drugs by pairs of diseases were calculated using bootstrapping (resampling with replacement) and, thus, are shown as average time difference ± standard deviation. Fig 3 and Fig 6 were rendered using iTOL [38].

## References

[1] Jordan VC. Tamoxifen: a most unlikely pioneering medicine. Nat Rev Drug Discov. 2003;2(3):205–213. 12612646 10.1038/nrd1031

[2] Radley DC, Finkelstein SN, Stafford RS. Off-label prescribing among office-based physicians. Arch Intern Med. 2006;166(9):1021–1026. 16682577 10.1001/archinte.166.9.1021

[3] Kesselheim AS. Rising health care costs and life-cycle management in the pharmaceutical market. PLoS Med. 2013;10(6):e1001461 10.1371/journal.pmed.1001461 23785261 PMC3681333

[4] Ashburn TT, Thor KB. Drug repositioning: identifying and developing new uses for existing drugs. Nat Rev Drug Discov. 2004;3(8):673–683. 15286734 10.1038/nrd1468

[5] Loging W, Rodriguez-Esteban R, Hill J, Freeman T, Miglietta J. Cheminformatic / bioinformatic analysis of large corporate databases: application to drug repurposing. Drug Discov Today. 2011;8(3–4):109–116.

[6] Davis AP, Wiegers TC, Roberts PM, King BL, Lay JM, Lennon-Hopkins K, et al A CTD-Pfizer collaboration: manual curation of 88,000 scientific articles text mined for drug-disease and drug-phenotype interactions. Database (Oxford). 2013;2013:bat080. 24288140 10.1093/database/bat080 PMC3842776

[7] Dudley JT, Deshpande T, Butte AJ. Exploiting drug-disease relationships for computational drug repositioning. Brief Bioinform. 2011;12(4):303–311. 10.1093/bib/bbr013 21690101 PMC3137933

[8] Hu G, Agarwal P. Human disease-drug network based on genomic expression profiles. PLoS One. 2009;4(8):e6536 10.1371/journal.pone.0006536 19657382 PMC2715883

[9] Nacher JC, Schwartz JM. A global view of drug-therapy interactions. BMC Pharmacol. 2008;8:5 10.1186/1471-2210-8-5 18318892 PMC2294115

[10] Dalkic E, Wang X, Wright N, Chan C. Cancer-drug associations: a complex system. PLoS One. 2010;5(4):e10031 10.1371/journal.pone.0010031 20368808 PMC2848862

[11] Zhao XM, Iskar M, Zeller G, Kuhn M, van Noort V, Bork P. Prediction of drug combinations by integrating molecular and pharmacological data. PLoS Comput Biol. 2011;7(12):e1002323 10.1371/journal.pcbi.1002323 22219721 PMC3248384

[12] Hoffmann R, Valencia A. Life cycles of successful genes. Trends Genet. 2003;19(2):79–81. 12547515 10.1016/s0168-9525(02)00014-8

[13] Pfeiffer T, Hoffmann R. Temporal patterns of genes in scientific publications. Proc Natl Acad Sci U S A. 2007;104(29):12052–12056. 17620606 10.1073/pnas.0701315104 PMC1924584

[14] Rzhetsky A, Wajngurt D, Park N, Zheng T. Probing genetic overlap among complex human phenotypes. Proc Natl Acad Sci U S A. 2007;104(28):11694–11699. 17609372 10.1073/pnas.0704820104 PMC1906727

[15] Loscalzo J, Kohane I, Barabasi AL. Human disease classification in the postgenomic era: a complex systems approach to human pathobiology. Mol Syst Biol. 2007;3:124 17625512 10.1038/msb4100163 PMC1948102

[16] Li Y, Agarwal P. A pathway-based view of human diseases and disease relationships. PLoS One. 2009;4(2):e4346 10.1371/journal.pone.0004346 19194489 PMC2631151

[17] Linghu B, Snitkin ES, Hu Z, Xia Y, Delisi C. Genome-wide prioritization of disease genes and identification of disease-disease associations from an integrated human functional linkage network. Genome Biol. 2009;10(9):R91 10.1186/gb-2009-10-9-r91 19728866 PMC2768980

[18] Stegmaier P, Krull M, Voss N, Kel AE, Wingender E. Molecular mechanistic associations of human diseases. BMC Syst Biol. 2010;4:124 10.1186/1752-0509-4-124 20815942 PMC2946303

[19] Rodriguez-Esteban R, Loging WT. Quantifying the complexity of medical research. Bioinformatics. 2013;29(22):2918–2924. 10.1093/bioinformatics/btt505 23995394

[20] Bohacek RS, McMartin C, Guida WC. The art and practice of structure-based drug design: a molecular modeling perspective. Med Res Rev. 1996;16(1):3–50. 8788213 10.1002/(SICI)1098-1128(199601)16:1<3::AID-MED1>3.0.CO;2-6

[21] Lipinski C, Hopkins A. Navigating chemical space for biology and medicine. Nature. 2004;432(7019):855–861. 15602551 10.1038/nature03193

[22] Glanville J, Zhai W, Berka J, Telman D, Huerta G, Mehta GR, et al Precise determination of the diversity of a combinatorial antibody library gives insight into the human immunoglobulin repertoire. Proc Natl Acad Sci U S A. 2009;106(48):20216–20221. 10.1073/pnas.0909775106 19875695 PMC2787155

[23] Deng ZL, Du CX, Li X, Hu B, Kuang ZK, Wang R, et al Exploring the biologically relevant chemical space for drug discovery. J Chem Inf Model. 2013;53(11):2820–2828. 10.1021/ci400432a 24125686

[24] Berglund L, Andrade J, Odeberg J, Uhlén M. The epitope space of the human proteome. Protein Sci. 2008;17(4):606–613. 10.1110/ps.073347208 18359855 PMC2271163

[25] Munos B. A forensic analysis of drug targets from 2000 through 2012 (2013) Clin Pharmacol Ther. 2013;94(3):407–411. 10.1038/clpt.2013.126 23756372

[26] Rogers EM. Diffusion of innovations New York: Free Press, 1995.

[27] Tufts Center for the Study of Drug Development. CNS Drugs Take Longer to Develop and Have Lower Success Rates than Other Drugs. November 4, 2014.

[28] Cokol M, Rodriguez-Esteban R. Visualizing evolution and impact of biomedical fields. J Biomed Inform. 2008;41(6):1050–1052. 10.1016/j.jbi.2008.05.002 18558511 PMC2650620

[29] Fournier JC, DeRubeis RJ, Hollon SD, Dimidjian S, Amsterdam JD, Shelton RC, et al Antidepressant drug effects and depression severity: a patient-level meta-analysis. JAMA. 2010;303(1):47–53. 10.1001/jama.2009.1943 20051569 PMC3712503

[30] Ebrahim S, Sohani ZN, Montoya L, Agarwal A, Thorlund K, Mills EJ, et al Reanalyses of randomized clinical trial data. JAMA. 2014;312(10):1024–1032. 10.1001/jama.2014.9646 25203082

[31] Arbesman S, Christakis NA. Eurekometrics: analyzing the nature of discovery. PLoS Comput Biol. 2011;7(6):e1002072 10.1371/journal.pcbi.1002072 21738456 PMC3127820

[32] Chiang AP, Butte AJ. Systematic evaluation of drug-disease relationships to identify leads for novel drug uses. Clin Pharmacol Ther. 2009;86(5):507–510. 10.1038/clpt.2009.103 19571805 PMC2836384

[33] Jung K, LePendu P, Chen WS, Iyer SV, Readhead B, Dudley JT, et al Automated detection of off-label drug use. PLoS One. 2014;9(2):e89324 10.1371/journal.pone.0089324 24586689 PMC3929699

[34] Vos R. Drugs looking for diseases. A descriptive model for the process of innovative drug research with special reference to the development of the beta blockers and the calcium antagonists [dissertation]. Groningen, Netherlands: University of Groningen, 1989.

[35] Thomas P, Starlinger J, Vowinkel A, Arzt S, Leser U. GeneView: a comprehensive semantic search engine for PubMed. Nucleic Acids Res. 2012;40(Web Server issue):W585–591. 10.1093/nar/gks563 22693219 PMC3394277

[36] Kuhn M, Szklarczyk D, Pletscher-Frankild S, Blicher TH, von Mering C, Jensen LJ, Bork P. STITCH 4: integration of protein-chemical interactions with user data. Nucleic Acids Res. 2014 1;42(Database issue):D401–7. 10.1093/nar/gkt1207 24293645 PMC3964996

[37] Myung IJ. Tutorial on maximum likelihood estimation. J Math Psychol. 2003;47:90–100.

[38] Letunic I, Bork P. Interactive Tree Of Life v2: online annotation and display of phylogenetic trees made easy. Nucleic Acids Res. 2011 7;39(Web Server issue):W475–8. 10.1093/nar/gkr201 21470960 PMC3125724

